# Benevolent Ageism’s Relationship to Self-Compassion and Meta-Memory in Older Adults

**DOI:** 10.1093/geroni/igaa057.1883

**Published:** 2020-12-16

**Authors:** Jennifer Sublett, Toni Bisconti

**Affiliations:** 1 University of Akron, Akron, Ohio, United States; 2 Univesity of Akron, Akron, Ohio, United States

## Abstract

Benevolence directed towards older adults can cross the line between respect and overaccommodation that undermines their physical and cognitive capabilities (Mehrotra & Wagner, 2009); however, little research has examined the subtleties of the influence of benevolent ageism on older adults’ ratings of their own functioning. Because stereotypes about older adults include the decline of mental abilities, this study examined whether their (N= 155) experiences with benevolent ageism, or overaccommodative offers of assistance and protection, influenced their own appraisals of memory abilities through their feelings of self-compassion. Older adults with fewer benevolent ageist experiences had higher rates of self-compassion, which in turn translated into better evaluations of their memory abilities. Future research should consider the potential pernicious influences that benevolent ageism has on older adults’ self-evaluations and performance, consider self-compassion as a buffer in these relationships, and test whether these relationships have downstream consequences on well-being outcomes.

